# Maximizing Data Quality using Mode Switching in Mixed-Device Survey Design: Nonresponse Bias and Models of Demographic Behavior

**DOI:** 10.12758/mda.2015.010

**Published:** 2015

**Authors:** William G. Axinn, Heather H. Gatny, James Wagner

**Affiliations:** University of Michigan Survey Research Center

**Keywords:** attrition, longitudinal (panel) study, mode switching, non-response bias, web survey, journal-keeping

## Abstract

Conducting survey interviews on the internet has become an attractive method for lowering data collection costs and increasing the frequency of interviewing, especially in longitudinal studies. However, the advantages of the web mode for studies with frequent re-interviews can be offset by the serious disadvantage of low response rates and the potential for nonresponse bias to mislead investigators. Important life events, such as changes in employment status, relationship changes, or moving can cause attrition from longitudinal studies, producing the possibility of attrition bias. The potential extent of such bias in longitudinal web surveys is not well understood. We use data from the Relationship Dynamics and Social Life (RDSL) study to examine the potential for a mixed-device approach with active mode switching to reduce attrition bias. The RDSL design allows panel members to switch modes by integrating telephone interviewing into a longitudinal web survey with the objective of collecting weekly reports. We found that in this design allowing panel members to switch modes kept more participants in the study compared to a web only approach. The characteristics of persons who ever switched modes are different than those who did not – including not only demographic characteristics, but also baseline characteristics related to pregnancy and time-varying characteristics that were collected after the baseline interview. This was true in multivariate models that control for multiple of these dimensions simultaneously. We conclude that mode options and mode switching is important for the success of longitudinal web surveys to maximize participation and minimize attrition.

## 1 Introduction

As internet access spreads worldwide, conducting survey interviews via the web has become an attractive method for lowering data collection costs while increasing the frequency of interviewing, especially in longitudinal studies (Couper, 2008). Web surveys are particularly appealing to researchers studying dynamic behaviors that require detailed, timing-specific measures collected over a long period of time (Axinn, Jennings & Couper, 2015; Stone, Shiffman, Atienza, & Nebeling, 2007; Mehl & Conner, 2013). Besides the cost savings, the advantages of web surveys for these studies include portability, flexibility, and confidentiality – web surveys allow respondents to complete surveys at whatever time and location is convenient and private for them. These properties extend to multiple devices including personal computers, laptops, tablets, and smartphones, further providing respondents with more options for convenience with little difference in measurement error between the devices (Lugtig & Toepoel, 2015). However, the advantages of the web mode for studies with frequent re-interviews can be offset by the serious disadvantage of low response rates and the potential for nonresponse bias to mislead investigators. Web surveys are known to have lower response rates compared to almost any other survey mode (Lozar Manfreda, Bosnjak, Berzelak, Haas, & Vehovar, 2008; Shih & Fan, 2008), and in longitudinal designs these lower response rates can produce serious misinformation regarding the true nature of changes over time (Graham & Donaldson, 1993; Lepkowski & Couper, 2002; Kristman, Manno, & Côté, 2005). In this paper we examine the potential for a mixed-device approach which allows panel members to switch modes – integrating telephone interviewing into a longitudinal web survey – to reduce the potential for attrition bias to produce misleading measures of dynamic behaviors.

We use data from the Relationship Dynamics and Social Life (RDSL) study, which was designed to investigate factors shaping the dynamics of sexual behavior, contraceptive use, and unintended pregnancy in a cohort of young adult women. The RDSL studied a random, population-based sample of 1,003 young women ages 18–19, residing in one county in the state of Michigan, USA. The representative sample of young women in the general population was accomplished by selection of individuals from the state driver’s license and personal identification card databases. Investigators conducted a 60-minute face-to-face baseline survey to launch the study and then enrolled women in a 2.5-year panel study that required completion of weekly surveys about contraceptive use, relationships, and prospective pregnancy intentions. Web and telephone modes were selected for the weekly surveys to maximize respondent privacy by eliminating the need for written records that must be kept and could potentially be discovered by a third party. Additionally, telephone surveys generally achieve higher response rates than either mail or web (Lozar, Manfreda, et al., 2008). Ninety-two percent of women in the baseline survey had internet access and were encouraged to complete the follow-ups surveys by web. Women without internet access were asked to complete the surveys by telephone. However, all women were provided the study website URL and telephone number and were allowed to complete each week’s survey by either mode. This protocol actively used mode switching to reduce non-response. Those who were late completing their journals were contacted by email first, then by phone, to complete their surveys. The face-to-face baseline interviews were conducted March 2008-July 2009 and the web-based panel study concluded in February 2012. The response rate for the baseline interview was 84% (RR1; AAPOR, 2011); 99% of those who completed the baseline survey agreed to participate in the panel; and 75% continued to participate in the panel for at least 18 months.

The RDSL design provides an unusually strong opportunity to investigate associations between individual characteristics measured in the baseline interview and subsequent participation in the panel study. Several studies have examined the consequences of changing modes on participation in a single wave in panel surveys (Jackle, Lynn, & Burton, 2015; Lynn, 2012; Hoogendoorn, Lamers, Penninx, & Smit, 2013; Wagner, Arrieta, Guyer, & Ofstedal, 2014). This study is unique in that interviewing was conducted weekly and panel members were allowed to switch between modes as necessary. This allows us to examine the impact on estimates of dynamic behaviors of allowing panel members to switch modes across multiple waves. In this paper we examine the extent to which use of a mixed-device approach and active mode-switching alter results relative to the alternative no-switching approach. Using RDSL measures we estimate the extent to which allowing mode-switching improves participation in the longitudinal measurement for select subgroups and characteristics. First, we use baseline measures to compare the cases who would have been represented if no mode-switching was allowed with the cases who remained in the study by allowing them to switch modes. Second, we use the baseline measures to assess associations between various individual characteristics and the number of mode switches each respondent made during the 18-month panel. Third, we investigate the extent to which the addition of the option to switch modes changes estimates of key behaviors in the panel study, including residential moves, changes in intimate partners, sexual experience, contraceptive use, and pregnancy. We also extend this investigation into estimates of consequences of specific intimate partner dynamics across the panel study to produce mode switching in subsequent journals. Finally, we investigate the extent to which key model parameters from previously published substantive results differ when models are estimated on cases that used the *same* mode for all interviews. Altogether the results provide important new evidence of the ability of mixed-device mode switching approaches to compensate for the weaknesses of single mode web-only approaches by reducing attrition.

## 2 Mixed-Device Mode Switching

Theoretically, allowing mixed-device mode switching in a panel design may have many advantages for maximizing participation across time. Two different processes define the total success maximizing survey participation: establishing contact with the respondent and the respondent’s consent to complete the survey. A crucial issue in obtaining respondent consent and cooperation is the incentive to burden ratio associated with completing the survey (Groves & Couper, 1998). Groves, Singer, and Corning (2000) describe this as the “leverage-saliency” theory of nonresponse. Survey respondents place different values on aspects of the survey request. Groves, Singer, and Corning label these “leverage.” Leverage can be either positive or negative. Some panel members place a high positive value on an incentive while others may be interested in completing the survey because they find the topic interesting. A long survey might be a negative leverage for some panel members. On the other hand, the survey design makes particular features of the design “salient.” For instance, the survey may emphasize the incentive or the interesting questionnaire in their contacts with panel members. Response rates are maximized when the appropriate set of design features are made salient to those for whom these features have larger leverage. For example, the shorter and easier a survey is to complete, the lower the negative leverage. For those panel members for whom this aspect of the survey is an important feature, making this salient may increase their probability of participating. Keeping survey tasks short always reduces the burden and this is especially important for repeated interviewing over time (longitudinal studies) and the more often the interview is repeated the more important this becomes. But different design features are salient for different respondents. One appeal of mixed-device surveys is the opportunity to allow each respondent to use whatever device is easiest for that respondent. With web surveys, computers, tablets, and smartphones could each be used, allowing each respondent to choose the device that is the least burden for that specific respondent. Allowing respondents to change devices across interviews provides the means for respondents to select the easiest device at each interview, enhancing the ease of the experiences. Easier experiences decrease negative leverage that may reduce the probability of completing the survey and thereby increase respondent participation.

Mode switching is a related design feature. Allowing the respondent to switch modes at each interview allows the respondent to select the easiest mode for the specific circumstances of that interview. Easier modes reduce burden and increase respondent cooperation. So dynamic life circumstances that make one mode easier one week and a different mode easier the next week support a design that allows mode switching to maximize respondent participation and reduce attrition. Residential moves, employment/financial change, or intimate partner changes are all examples of factors likely to make mode switching appealing. In fact, life circumstances that make daily activities more complicated in any way, including pregnancy, childbirth, poverty, traumatic experience, health limitation, or other crisis circumstances all make ease of completing the survey a high priority in maintaining high respondent cooperation. To the extent mode-switching makes completing the survey easier, any of these circumstances may motivate mode switching as a means to increase participation and reduce attrition.

Mode switching may be equally valuable for establishing contact with respondents across multiple interviews in a longitudinal survey. A key source of attrition in longitudinal surveys is failure to re-contact the specific respondent at future interviews (Groves & Couper, 1998; Schoeni, Stafford, McGonagle, & Andreski, 2013; Couper and Ofstedal, 2009; Ribisl et al., 1996). Many factors make failure to re-contact likely, especially residential moves, but also job loss, divorce, intimate partner breakups, and significant income changes (Lepkowski & Couper, 2002; Trappmann, Gramlich, & Mosthaf, 2015). Life changes that make it more difficult to locate respondents or find them available to complete a survey may reduce re-contact. The portability of both web and phone make them desirable modes in these circumstances, but the ability to switch across these modes may enhance the overall ease of responding. Thus longitudinal surveys that provide mode-switching options may be more successful at keeping respondents with complex or changing life circumstances involved in longitudinal surveys.

## 3 Data, Mode Switching Measures, and Analysis Plan

### 3.1 Data

The Relationship Dynamics and Social Life (RDSL) study focuses on 18–19 year old women in a single county in the State of Michigan, USA. The specific county was selected both because several key demographic characteristics of that county fell near the median for the State and because the county had a high degree of variability with respect to income and race, providing high diversity in the general population without requiring over-samples of sub-groups (Barber, Kusunoki, & Gatny, 2011). Sixty-minute face-to-face baseline interviews were conducted with each woman at the start of the study to gather information on her family background; education and career plans; attitudes, values, beliefs, and knowledge about sexual practices; romantic relationships; and sexual experiences. After the baseline interview, the women were each invited to participate in the weekly journal portion of the study. Over 99% of respondents who completed the baseline survey enrolled in the weekly surveys (n=992) (Barber et al., 2011).

Significant effort was taken to keep these young women enrolled in the weekly journal-keeping study. The burden of each weekly interview was kept low by maintaining an average interview length of seven minutes or less. Emails and/or text messages were sent weekly to remind respondents. Monetary incentives of $1 per weekly journal and a bonus of $5 for having completed five weekly journals on time were given, and small gifts—such as pens and lip balm—were also given to encourage retention (Gatny, Couper, Axinn, & Barber, 2009). Respondents who failed to complete the journal on time were contacted by email and phone, and then eventually by letter. After 60 days of not completing a journal, increased incentives were offered for the next journal entry.^1^ At the completion of the journal-keeping study, 84% of respondents who were interviewed at baseline had participated in journal-keeping for at least 6 months, 79% for at least 12 months, and 75% for at least 18 months with some journals missing (Barber et al., 2011).

### 3.2 Measures of Mode Switching

For this study of mode-switching, we confine our analyses to the 947 respondents who completed 2 or more journals. We analyze journals completed within the first 18 months of journal enrollment (n=39,598) to minimize bias from attrition. At baseline 92% (872/947) of respondents selected to complete the journals by web and 8% (75/947) selected the phone instead. Of the 872 respondents who selected the web, 60% (520/872) completed at least one journal by phone. The range was 1–78 journals completed by phone among these respondents who initially selected the web, and the mean was 8 journals completed by phone. Note this count does not include the mode for journal 1 because that journal was completed with the interviewer.

Of the 75 respondents who selected the phone, 39% (29/75) completed at least one journal by web. The range was 1–64 journals completed by web among these respondents who initially selected the phone, and the mean was 23 journals completed by web. Again this count does not include the mode for journal 1 because that journal was completed with the interviewer.

To construct a measure of the count of the number of mode switches which took place we created a variable counting the number of times a respondent completed a journal in a mode different from the mode used at the previous journal. Note this measure does not include journal 1 in the count because that journal was unlike all others – it was conducted during the baseline interview with the interviewer. This measure also does not include journal 2 because it is the first journal that the respondent completed without the help of the interviewer. Also, a large proportion (84% or 132/157) of those who only had one mode switch had the switch at journal 2. In other words, they did journal 2 in a mode different than what they enrolled in at baseline. The measure of the number of mode switches begins counting switches at journal 3 (n=37,659). Starting at journal 3, a switch is a mode different from the mode used at the previous journal.

The range of mode switching was 0–30 switches. More than half of the sample (504 respondents) had zero mode switches. Though this is a large group of stable single mode users, nearly half of the sample (443 respondents) had at least one mode switch. The mean number of switches was 1.93 and the most common number of switches was two. Over 16% of the sample experienced two mode switches – two switches implies starting in one mode, completing a single journal in the alternate mode, and then returning to the initial mode for the remainder of the study. Nearly 25% of the sample experienced three or more mode switches.

The timing of mode switching as respondents complete more journals implies some switching motivated by the respondent’s experience with the initial mode. For example, 29% (18/62) of those who only had one mode switch had the switch at journal 3. Journal one was completed with the interviewer, journal 2 was the respondent’s first journal alone, and the journal 3 switches took place during the respondent’s second interview alone. In other words, they completed that journal in a mode different than what they used at journal 2, the first journal completed without the help of an interviewer present. Some may have simply wanted to try an alternative to see if it was easier, others may have had a negative experience with their first attempt to complete the journal on their own. Respondents experienced their first mode switch across journals 3 through 71, but by journal 8, more than half of respondents (228/443) who ever experienced a switch had experienced their first switch. Over the 18 months analyzed here respondents could have completed as many as 78 journals, but first mode switches appear to take place early in the process.

### 3.3 Analysis Strategy

Our analysis proceeds in three steps, each time focusing on mode-switching as the key alternative to attrition from the study. In the first step we use data from the baseline interview before the weekly journal keeping is launched to assess the associations between baseline characteristics and mode switching behavior. This analysis has two parts. In part one we use the comparison of those cases who only used a single mode to those cases who remained in the RDSL by switching modes to perform t-tests of mean differences, allowing us to identify prior characteristics associated with subsequent mode switching. In part two we estimate multivariate models of the likelihood of ever making a mode switch and of the number of mode switches. This part of this step allows us to assess the independence of associations between various prior background characteristics and respondents’ mode switching behaviors.

In the second step we use data from the journal itself to assess the causes and consequences of mode switching rather than attrition from the study. Again, this analysis has two parts. First, we investigate the overall relationship between mode switching behavior and other behaviors reported in the journal. Here we compare key behaviors measured in the journal between those cases who only used a single mode and those cases who remained in the study by switching modes. Second, we investigate the association between measures of weekly relationship dynamics and the likelihood the week ends in an interview mode switch. The investigation uses the special relationship dynamics measures from the RDSL study to highlight how those behaviors themselves may be associated with mode switching.

In the third step, we assess the extent to which substantive conclusions from multivariate models can be altered by eliminating the mode switching alternative to attrition from the study. We use a specific model previously published using RDSL data. We estimate this model as published, and then re-estimate the model assuming the cases that used mode switching would have dropped out of the study (attrition). This comparison highlights the potential substantive research consequences of allowing interview mode switching as an alternative to attrition from the study.

## 4 Results

### 4.1 Baseline Characteristics and Subsequent Mode Switches

#### 4.1.1 Comparison of respondents who switch mode with those who do not

Our analysis begins with comparisons between those who switched modes during the 18-month panel study and those who did not (Table 1). We present three versions of each statistic – one for the total sample, one for those who never switched modes, and one for those who ever switched modes (Table 1). Those who switched modes are the most likely to be lost to attrition in a single mode study. The p-values associated with each row indicate the statistical significance of the difference in each statistic between the respondent who never switched modes and those who ever switched modes. For example, there is a statistically significant difference in mode switching with African Americans being more likely to switch at least one time (row 1 of Table 1), but there is not a significant difference in high-school grade point average (GPA) between those who switched modes and those who did not (row 3 of Table 1). Overall, there are many statistically significant differences in key statistics displayed in Table 1.

We group respondent characteristics into four domains – Sociodemographic Characteristics, Childhood Family Background Measures, Childhood Socioeconomic Status, and Experiences Related to Pregnancy (the main substantive topic of RDSL). All of these characteristics were measured during the baseline interview, before journal-keeping began. None of the measures of experiences related to pregnancy are associated with mode switching during the panel study. By contrast, measures in each of the other domains are associated with significant differences in switching behavior, or potential attrition if switching was not allowed.

Among sociodemographic characteristics, both being African American (compared to being white) and receiving public assistance are associated with significantly higher likelihood of mode switching. We argue any life circumstances that create complexity of social experience are likely to be associated with higher likelihood of mode switching – both results are consistent with that argument. Being enrolled in school full-time is associated with significantly lower likelihood of mode switching. This result is consistent with full-time school promoting stability of experience in early adulthood, in contrast to either part-time or no school. Early adult income levels and car ownership are not significantly associated with mode switching.

Within the domain of childhood family background, growing up in a two-parent family is associated with a significantly lower likelihood of mode switching during the panel study. High religiosity in the childhood family of origin is associated with significantly higher likelihood of mode switching. Experiencing a relatively young mother is not associated with subsequent mode switching. Within the domain of childhood socioeconomic status, growing up in a household that received public assistance is associated with a significantly higher likelihood of mode switching. Growing up in a household in which parents owned their own home or had high incomes were both associated with significantly lower likelihood of mode switching during the panel study. Growing up with parents who had at least some college education is not associated with subsequent mode switching. Again, factors associated with higher mode switching would likely produce attrition if the mode alternatives were not provided.

This initial step in our analysis examines only bivariate associations. In the next step we move on to multivariate models of ever making a mode switch and the number of mode switches – this step allows us to assess the independences of these various associations between individual respondent background and mode switching behaviors.

#### 4.1.2 Associations between respondent background and both likelihood of mode switch and numbers of mode switches

Using the same background characteristics as presented in Table 1, we now estimate multivariate models of mode switching behavior (likelihood of attrition under a single mode design). The first column of Table 2 presents results from a logistic regression model using all the characteristics to predict the likelihood the respondent makes any mode switch. Significant associations documented in this column indicate the specific characteristic is associated with making a mode switch independent of the other bivariate associations documented in Table 1. Among these characteristics, being African American, enrolled in school full-time, from a two-parent family, or having parents who owned their own home, each has an independent statistically significant association with ever switching interview modes during the panel study (column 1, Table 2). This means that panel studies of this type which do not allow mode switching may underrepresent respondents who are African American, who are not enrolled in school full-time, who do not come from a two-parent family, and who have parents who did not own their own home. Such attrition bias has the potential to undermine substantive results based on studies that do not allow mode switching. Finally note that in these multivariate models we also control for the length of time in the study before the mode switch – remaining in the study longer significantly increases the likelihood of a mode switch. Consistent with predictions, efforts to keep respondents in longitudinal panel studies for longer periods of time will be more successful when mode switching is designed into the data collection.

Next we use the same measures of respondent background to estimate models of the number of times each individual switches interview modes. Here we use Poisson regression (column 2 of Table 2) because the high skew in the count measure fits a Poisson distribution. The distributional assumptions of the Poisson regression are more consistent with this count of number of switches. This is important because the results in column 2 of Table 2 demonstrate that the majority of background characteristics we measure (11 of 19) have statistically significant and independent associations with the number of mode switches a respondent makes during the 18-month panel study. Failure to allow mode switching in such a panel study greatly increases the chance that the resulting measures will be selective on many different dimensions of social life.

### 4.2 Journal Measures and Journal Mode Switching

#### 4.2.1 Comparing journal measures for those who switched modes and those who did not

Next we examine data from the journal itself. We begin by comparing reports of key substantive behaviors measured in RDSL between respondents who never switched modes and respondents who ever switched modes. The behaviors we investigate include if the respondent received public assistance, changed residence, had sex, had sex without contraception, had sex with a new partner, had sex with more than one partner, had conflict with a partner, lived with a partner, or became pregnant. Table 3 summarizes our findings.

The p-value indicated in each row describes the statistical significance of each comparison. All of these comparisons are statistically significant and in every case the sample who experienced a mode switch had a higher value on the measures. This table provides a powerful summary of the importance of mode switching. In every type of behavior representing core domains of this study, mode switching was associated with higher levels. Without allowing mode switching, it appears the RDSL study would have significantly underestimated each and every core behavior the study was designed to measure.

#### 4.2.2 Predicting mode switches from key behaviors

Now we investigate the possibility that the core behaviors themselves motivate a mode switch. Behaviors such as change in intimate partner relationship status are believed to increase attrition from longitudinal studies because they make locating respondents and convincing those respondents to participate more difficult. Here we use the weekly behaviors of participants in the RDSL study to predict the chances they end the week with a mode switch. Because receiving public assistance was only measured in RDSL quarterly and place of residence was only measured in RDSL monthly, we do not investigate these two factors. Instead we focus on the weekly dynamics of relationships, including sex, contraception, conflict, and pregnancy. In each case we estimate both a bivariate association and then we re-estimate that association controlling for the full set of baseline interview characteristics we examined earlier. The results are presented in Table 4.

Each column of Table 4 comes from a separate model estimate. In columns 5, 7, and 9 of Table 4 we see that sex without contraception, sex with a new partner, and sex with a second partner are each significantly associated with a mode switch at the end of the week, independent of key baseline characteristics. These events increase the likelihood of a mode switch; these data provide evidence that some sexual events may lead to mode switching in the short term. Single mode studies would likely lose respondents who had just experienced similar events, biasing reports of such events downward.

### 4.3 Substantive Model with and without Mode-Switching

In this analysis (Table 5), we investigate the potential impact of not allowing mode switching on a multivariate model developed to investigate the impact of ambivalent fertility desires on pregnancy risk (Miller, Barber, & Gatny, 2012 {Table 3, Column 3}). This model included a number of demographic control variables as well as experiences related to pregnancy from the baseline interview, such as being 16 years of age or less at first sex. The model, as reported in published research, includes all of the available data. In this original, published model, the desire to become pregnant is a significant and positive predictor of the probability of actually becoming pregnant. Further, the desire to avoid pregnancy is a significant, independent, and negative predictor of the probability of becoming pregnant. This result provided empirical evidence of the simultaneous influence of contrasting attitudes toward pregnancy – an important theoretical advance in our understanding of the relationship among attitudes, intentions, and young adult pregnancies.

The substantive conclusions from the original estimated model are substantially changed when data collected after the first mode switch are omitted. Had mode switching not been an option, many in the study would have likely stopped providing measures (attrition). When the data these respondents provided after the mode switch are excluded, the originally significant relationships, although similar to the originally estimated effects, change in ways that would alter substantive conclusions. For example, comparing row one across the two models, the size of the association with desire to become pregnant drops by more than 20% and is no longer statistically significantly different from zero association. Had the model been estimated on these truncated data, estimates would not have provided any empirical support for substantive conclusions that contrasting attitudes may simultaneously shape behavioral choices in opposing directions.

Some of these differences are due to sampling error. The number of journals included in the original model was 34,377. After excluding journals that were completed after the first mode switch, there were 21,573 completed journals. The other explanation for the changed estimates is the changing composition of the response. For example, we see a change in the estimate of the coefficient for cohabiting, which is now significant after deleting journals collected after the first mode switch. Further, some of the baseline characteristics related to pregnancy that were only marginally significant in the original model are now significant in the model on the subset of journals collected before the first mode switch. These include receiving public assistance and the number of previous pregnancies.

For this published model, the data collection strategy allowing respondents to switch modes at multiple points in the data collection process prevent attrition among enough respondents to make a difference in substantive conclusions. Although some of these differences are related to a reduction in sample size, which would likely occur under a single mode strategy, others are due to the composition of who responds when mode switching is available to avoid attrition.

## 5 Discussion

We know from previous research that attrition from panel studies can be caused by important life events, such as changes in employment status, relationships, or moving (Lepkowski & Couper, 2002; Trappmann, et al., 2015). When these events are the topic of the study, this attrition can lead to significant attrition related bias. The potential extent of such bias in studies featuring frequent measurement to document rapidly changing attitudes and behaviors (such as RDSL) is not well understood.

We found that in a panel survey that collects data weekly, allowing panel members to switch modes was an important approach for reducing attrition bias. The characteristics of persons who ever switched modes are different – including not only demographic characteristics, but also baseline characteristics related to pregnancy and time-varying characteristics that were collected after the baseline interview. This was true even for multivariate models that control for many of these dimensions. The fact that the data from the journal predicts whether or not a mode switch was made is a strong indication that estimates that are based on a procedures that do not allow respondents to switch modes would be characterized by attrition bias.

Of course all studies have limitation, including the one we report here. This study focused on women only and focused on women in a narrow age range. Although the results cannot be extrapolated to men or those at older ages, it is quite likely that many of the same issues apply. The longitudinal study described here featured weekly measurement – longitudinal studies with less frequent interviewing may not be able to use mode switching to reduce attrition as effectively. The study reported here also focused on relationships, sex, contraception, and pregnancy – again it is possible that studies of other topics show fewer potential effects of attrition from failure to allow mode switches. Nevertheless, it is quite likely the same issues described here face longitudinal studies of most topics. From the results presented above, we conclude that not allowing users to switch modes in studies with frequent measurement of attitudes or behaviors increases the risk of attrition bias in estimates.

Our research suggests that it may be possible to profile panel members using data from the baseline interview in order to identify cases for whom mode switching may be an effective tool for combating attrition. Lugtig, for example, uses a factor analysis to define profiles of classes of attriters (2014). Armed with early predictions of which cases may fit the profile of “mode-switchers,” survey designers may deploy an “adaptive” design (Wagner, 2008; Schouten & Calinescu, 2011) that tailors the survey design to the characteristics of the sampled unit. In this case, the goal of this design would be to prevent attrition bias.

Web surveys are particularly appealing to researchers studying dynamic behaviors that require detailed, timing-specific measures collected over a long period of time (Axinn et al., 2015; Stone et al., 2007; Mehl & Conner, 2013). Besides the cost savings, the advantages of web surveys for these studies include portability, flexibility, and confidentiality – web surveys allow respondents to complete surveys at whatever time and location is convenient and private for them. These properties extend to multiple devices including personal computers, laptops, tablets, and smartphones, further providing respondents with more options for convenience with little difference in measurement error between the devices (Lugtig & Toepoel, 2015). Even though web surveys are known to have lower response rates compared to almost any other survey mode (Lozar Manfreda et al., 2008; Shih & Fan, 2008), in this paper we demonstrate the potential for a mixed-device approach to compensate for this weakness and strengthen the web survey approach for frequent, repeated measurement. The approach we advocate allows panel members to switch modes – integrating telephone interviewing into a longitudinal web survey – to reduce the potential for attrition bias to produce misleading measures of dynamic behaviors. Overall, the mixed-device approach brings respondents into the study who are significantly different, making conclusions from the mixed-device panel study more robust.

Previously published methodological results from the special RDSL mixed-mode panel are complementary to the results we present here, all indicating this important tool has many advantages. Other investigations of the method not only provide more detailed descriptions of the study (Barber et al. 2011), but also demonstrate that frequent interviewing does not bias measures (Axinn et al. 2015; Barber, Gatny, Kusunoki, & Schulz, Forthcoming), that the web-phone mix has the potential for integrated biomarker collection (Gatny, Couper, & Axinn, 2013), and that the use of electronic debit cards to pay respondent incentives can greatly enhance the feasibility of this approach (Gatny et al. 2009). Overall this body of methodological research demonstrates many advantages of the mixed-mode, mixed device RDSL approach to frequent repeated survey measurement.

## Figures and Tables

**Table 1:** Respondent characteristics

	Total Sample (n=947) %	Subsample who used same mode at every journal (n=504) %	Subsample with at least one mode switch (n=443) %	*p*-value
**Sociodemographic Characteristics**				
African American	.34	.28	.41	***
Enrolled in school full-time	.51	.54	.47	*
High school GPA a	3.12^b^	3.15	3.09	
<$1,000 (1st quartile)	.35	.33	.37	
Currently receiving public assistance	.26	.24	.29	+
Income not enough to make ends meet	.18	.17	.20	
Owns a car	.48	.50	.46	
**Childhood Family Background Measures**				
Two-parent childhood family structure	.52	.58	.46	***
Biological mother <20 years old at 1st birth	.37	.35	.38	
High religiosity	.57	.54	.61	*
**Childhood Socioeconomic Status**				
Received public assistance	.37	.33	.41	**
At least one parent has at least some college	.66	.68	.64	
Parents were home owners	.71	.75	.65	***
High parent income	.38	.42	.33	**
**Experiences Related to Pregnancy**				
Living with partner	.17	.17	.17	
Age at first sex ≤ 16 years	.51	.49	.54	
Two or more sexual partners	.60	.58	.61	
Ever had sex without birth control	.48	.46	.50	
1 or more prior pregnancies	.15	.14	.16	

+ p < 0.10;

* p < 0.05;

** p < 0.01;

*** p < 0.001 (two-tailed independent samples t-tests for significant differences between the two subsamples)

a mean GPA presented for sample and subsamples;

b std. dev.=.61

**Table 2:** Regression coefficients for models of at least one journal mode switch (logistic) and number of journal mode switches (poisson) (N=947)

	(1) at least one journal mode switch	(2) number of journal mode switches
**Sociodemographic characteristics**		**POISSON**
African American	**.53** ** **(.18)**	**.39** *** **(.06)**
Enrolled in school full-time	**−.29** ^+^ **(.15)**	**−.28** *** **(.05)**
High school GPA	−.13 (.13)	−.06 (.04)
<$1,000 (1st quartile)	.06 (.16)	**.16** ** **(.05)**
Currently receiving public assistance	.08 (.19)	**.20** *** **(.06)**
Income not enough to make ends meet	.03 (.19)	**.15** * **(.06)**
Owns a car	.15 (.15)	**−.14** ** **(.05)**
**Childhood family background measures**		
Two-parent childhood family structure	**−.31** ^+^ **(.16)**	−.04 (.05)
Biological mother <20 years old at 1st birth	−.03 (.16)	.05 (.05)
High religiosity	.08 (.15)	.08 (.05)
**Childhood socioeconomic status**		
Received public assistance	.09 (.17)	.09 (.05)
At least one parent has at least some college	.00 (.16)	**.16** ** **(.05)**
Parents were home owners	**−.33** ^+^ **(.18)**	**−.21** *** **(.05)**
High parent income	−.06 (.17)	**−.24** *** **(.06)**
**Experiences related to pregnancy**		
Living with partner	.01 (.21)	−.05 (.07)
Age at first sex < 16 years	.11 (.19)	**.13** * **(.06)**
Two or more sexual partners	.00 (.19)	.02 (.06)
Ever had sex without birth control	.04 (.18)	.06 (.06)
1 or more prior pregnancies	.10 (.22)	**−.27** *** **(.07)**
**Other**		
Time in study	**.13** *** **(.02)**	**.13** *** **(.01)**
χ^2^	120.49	
Pseudo-R^2^	.09	
R^2^		.15

Standard errors in parentheses.

† p < 0.10;

* p < 0.05;

** p < 0.01;

*** p < 0.001 (two-tailed tests)

**Table 3:** Respondent behaviors reported in the journal

	Total Sample (n=947) %	Subsample who used same mode at every journal (n=504) %	Subsample with at least one mode switch (n=443) %	*p*-value
Received public assistance	.25	.19	.32	***
Changed residence	.40	.33	.49	***
Sex	.78	.73	.82	**
Sex without contraception	.50	.41	.59	***
Sex with a new partner	.45	.38	.52	***
Sex with someone other than current partner	.18	.13	.24	***
Conflict with a partner	.16	.11	.21	***
Lived with a partner	.41	.35	.48	***
Pregnant	.13	.10	.18	***

+ p < 0.10;

* p < 0.05;

** p < 0.01;

*** p < 0.001 (two-tailed independent samples t-tests for significant differences between the two subsamples)

**Table 4:** Logistic regression coefficients for models using behaviors reported in a journal to predict a mode switch in the same journal (N=37,659)

	1	2	3	4	5	6	7	8	9	10	11	12	13	14	15
	
**Behavior reported in the journal**															
Sex		.16 ^+^ (.09)	.10 (.09)												
Sex without contraception				.50 *** (.11)	.22 * (.11)										
Sex with a new partner						.56 *** (.14)	.44 ** (.14)								
Sex with someone other than current partner								.54 ** (.19)	.31 ^+^ (.18)						
Conflict with a partner										.38 ^+^ (.22)	.09 (.23)				
Living with a partner												.10 (.12)	−.07 (.15)		
New pregnancy														.64 * (.31)	.19 (.31)
**Sociodemographic characteristics**															
African American	.48 *** (.13)		.49 *** (.13)		.48 *** (.13)		.48 *** (.13)		.48 *** (.13)		.48 *** (.13)		.47 *** (.13)		.48 *** (.13)
Enrolled in school full-time	−.22 * (.10)		−.21 * (.10)		−.20 * (.10)		−.21 * (.10)		−.21 * (.10)		−.22 * (.10)		−.22 * (.10)		−.22 * (.10)
High school GPA	−.18 ^+^ (.09)		−.18 ^+^ (.09)		−.17 ^+^ (.09)		−.18 ^+^ (.09)		−.18 ^+^ (.09)		−.18 ^+^ (.09)		−.18 ^+^ (.09)		−.18 ^+^ (.09)
<$1,000 (1st quartile)	.12 (.11)		.12 (.11)		.12 (.11)		.12 (.11)		.12 (.11)		.12 (.11)		.12 (.11)		.12 (.11)
Currently receiving public assistance	.27 ^+^ (.15)		.28 ^+^ (.15)		.28 ^+^ (.15)		.27 ^+^ (.15)		.27 ^+^ (.15)		.27 ^+^ (.15)		.27 ^+^ (.15)		.27 ^+^ (.15)
Income not enough to make ends meet	.19 (.14)		.19 (.14)		.18 (.14)		.19 (.14)		.19 (.14)		.19 (.14)		.19 (.14)		.19 (.14)
Owns a car	−.10 (.12)		−.11 (.12)		−.10 (.12)		−.10 (.12)		−.10 (.12)		−.10 (.12)		−.10 (.12)		−.10 (.12)
**Childhood family background measures**															
Two-parent childhood family structure	−.09 (.12)		−.09 (.13)		−.09 (.12)		−.09 (.12)		−.09 (.12)		−.09 (.12)		−.09 (.12)		−.09 (.12)
Biological mother <20 years old at 1st birth	.11 (.12)		.11 (.12)		.10 (.12)		.11 (.12)		.11 (.12)		.11 (.12)		.11 (.12)		.11 (.12)
High religiosity	.13 (.12)		.13 (.12)		.13 (.12)		.13 (.12)		.13 (.12)		.13 (.12)		.13 (.12)		.13 (.12)
**Childhood socioeconomic status**															
Received public assistance	.11 (.13)		.11 (.13)		.10 (.13)		.11 (.13)		.11 (.13)		.11 (.13)		.11 (.13)		.11 (.13)
At least one parent has at least some college	.08 (.12)		.08 (.12)		.08 (.12)		.08 (.12)		.08 (.12)		.08 (.12)		.08 (.12)		.08 (.12)
Parents were home owners	−.21 (.13)		−.21 (.13)		−.22 ^+^ (.13)		−.21 (.13)		−.21 (.13)		−.21 (.13)		−.21 (.13)		−.21 (.13)
High parent income	−.26 * (.13)		−.26 * (.13)		−.25 * (.13)		−.26 * (.13)		−.26 * (.13)		−.26 * (.13)		−.26 * (.13)		−.26 * (.13)
**Experiences related to pregnancy**															
Living with partner	−.01 (.17)		−.03 (.17)		−.03 (.17)		.00 (.17)		−.01 (.17)		−.01 (.17)		.03 (.19)		−.01 (.17)
Age at first sex ≤ 16 years	.14 (.16)		.14 (.16)		.14 (.16)		.14 (.16)		.14 (.16)		.14 (.16)		.15 (.16)		.14 (.16)
Two or more sexual partners	.10 (.16)		.08 (.16)		.08 (.16)		.09 (.16)		.10 (.16)		.10 (.16)		.10 (.16)		.10 (.16)
Ever had sex without birth control	.12 (.14)		.11 (.14)		.11 (.14)		.12 (.14)		.12 (.14)		.12 (.14)		.13 (.14)		.12 (.14)
1 or more prior pregnancies	−.21 (.16)		−.22 (.16)		−.21 (.16)		−.21 (.16)		−.21 (.16)		−.21 (.16)		−.21 (.16)		−.21 (.16)
**Other**															
Time in study	−.04 *** (.01)	−.04 *** (.01)	−.04 *** (.01)	−.04 *** (.01)	−.04 *** (.01)	−.04 *** (.01)	−.04 *** (.01)	−.04 *** (.01)	−.04 *** (.01)	−.04 *** (.01)	−.04 *** (.01)	−.04 *** (.01)	−.04 *** (.01)	−.04 *** (.01)	−.04 *** (.01)

χ^2^	241.22	34.05	245.45	56.02	255.41	49.03	255.76	42.13	248.71	35.29	243.22	32.66	242.39	36.33	242.55
Log-likelihood	−6933.04	−7279.52	−6931.28	−7260.13	−6928.25	−7276.90	−6928.08	−7281.13	−6931.77	−7282.81	−6932.93	−7283.82	−6932.65	−7283.07	−6932.87

Standard errors in parentheses.

† p < 0.10;

* p < 0.05;

** p < 0.01;

*** p < 0.001 (two-tailed tests)

**Table 5:** Logistic regression estimates of the effects of positive and negative pregnancy desires on the hazard of pregnancy

	Original Model	Subsample without mode switches
Desire to become pregnant	.22 * (.10)	.17 (.12)
Desire to avoid pregnancy	−.24 ** (.09)	−.26 * (.10)
**Sociodemographic characteristics**		
African American	.25 (.26)	.46 (.36)
Enrolled in school full time	−.15 (.22)	−.01 (.32)
Graduated high school	.36 (.25)	.46 (.35)
Receiving public assistance	.43 ^+^ (.25)	.63 * (.32)
Importance of Religion	.21 (.13)	.22 (.17)
Biological mother <20 years old at first birth	.19 (.22)	−.09 (.29)
One biological parent only (ref=2 parents)	.06 (.25)	−.13 (.33)
Other (ref=2 parents)	.16 (.36)	.32 (.46)
Mother‘s education <high school graduate	.09 (.34)	−.37 (.56)
$15,000–44,999 (ref<=14,999)	−.60 * (.31)	−.87 * (.42)
$45,000–74,999 (ref<=14,999)	−.68 ^+^ (.38)	−.47 (.50)
$75,000 or greater (ref<=14,999)	−.56 (.43)	−.51 (.53)
Don‘t know/refused (ref<=14,999)	−.34 (.30)	−.26 (.39)
Age at first sex 16 years or less	.67 * (.30)	.36 (.40)
Lifetime number of sexual partners two or more	.70 * (.31)	.48 (.40)
Ever had sex without birth control	.23 (.27)	.38 (.38)
Number of previous pregnancies	.17 ^+^ (.10)	.24 * (.12)
Cohabiting	.38 (.24)	.87 ** (.32)
Age	−.26 (.20)	−.17 (.26)
**Other**		
Time-to-pregnancy	.29 *** (.08)	.30 * (.12)
Time-to-pregnancy squared	−.01 ** (.00)	−.02 * (.01)
Number of journals	−.02 *** (.00)	−.02 *** (.00)
χ^2^	174.93	133.97
Log likelihood	−700.58	−340.69
Pseudo-R^2^	.14	.17
Journal N	34,377	21,573
Respondent N	887	758

Standard errors in parentheses.

† p < 0.10;

* p < 0.05;

** p < 0.01;

*** p < 0.001 (two-tailed tests)

## Footnotes

1 See Barber et al. (2012) for more information on the design and implementation of the RDSL study.
